# Deep learning–based metal artefact reduction in PET/CT imaging

**DOI:** 10.1007/s00330-021-07709-z

**Published:** 2021-02-10

**Authors:** Hossein Arabi, Habib Zaidi

**Affiliations:** 1 Division of Nuclear Medicine and Molecular Imaging, Geneva University Hospital, CH-1211 Geneva 4, Switzerland; 2 Geneva University Neurocenter, Geneva University, CH-1205 Geneva, Switzerland; 3 Department of Nuclear Medicine and Molecular Imaging, University of Groningen, University Medical Center Groningen, 9700 RB Groningen, the Netherlands; 4 Department of Nuclear Medicine, University of Southern Denmark, DK-500 Odense, Denmark

**Keywords:** Positron emission tomography, Computed X-ray tomography, Artefacts, Deep learning, Artificial intelligence

## Abstract

**Objectives:**

The susceptibility of CT imaging to metallic objects gives rise to strong streak artefacts and skewed information about the attenuation medium around the metallic implants. This metal-induced artefact in CT images leads to inaccurate attenuation correction in PET/CT imaging. This study investigates the potential of deep learning–based metal artefact reduction (MAR) in quantitative PET/CT imaging.

**Methods:**

Deep learning–based metal artefact reduction approaches were implemented in the image (DLI-MAR) and projection (DLP-MAR) domains. The proposed algorithms were quantitatively compared to the normalized MAR (NMAR) method using simulated and clinical studies. Eighty metal-free CT images were employed for simulation of metal artefact as well as training and evaluation of the aforementioned MAR approaches. Thirty ^18^F-FDG PET/CT images affected by the presence of metallic implants were retrospectively employed for clinical assessment of the MAR techniques.

**Results:**

The evaluation of MAR techniques on the simulation dataset demonstrated the superior performance of the DLI-MAR approach (structural similarity (SSIM) = 0.95 ± 0.2 compared to 0.94 ± 0.2 and 0.93 ± 0.3 obtained using DLP-MAR and NMAR, respectively) in minimizing metal artefacts in CT images. The presence of metallic artefacts in CT images or PET attenuation correction maps led to quantitative bias, image artefacts and under- and overestimation of scatter correction of PET images. The DLI-MAR technique led to a quantitative PET bias of 1.3 ± 3% compared to 10.5 ± 6% without MAR and 3.2 ± 0.5% achieved by NMAR.

**Conclusion:**

The DLI-MAR technique was able to reduce the adverse effects of metal artefacts on PET images through the generation of accurate attenuation maps from corrupted CT images.

**Key Points:**

• *The presence of metallic objects, such as dental implants, gives rise to severe photon starvation, beam hardening and scattering, thus leading to adverse artefacts in reconstructed CT images.*

• *The aim of this work is to develop and evaluate a deep learning–based MAR to improve CT-based attenuation and scatter correction in PET/CT imaging.*

• *Deep learning–based MAR in the image (DLI-MAR) domain outperformed its counterpart implemented in the projection (DLP-MAR) domain. The DLI-MAR approach minimized the adverse impact of metal artefacts on whole-body PET images through generating accurate attenuation maps from corrupted CT images.*

**Supplementary Information:**

The online version contains supplementary material available at 10.1007/s00330-021-07709-z.

## Introduction

A large number of patients referred for whole-body PET/CT examinations present with metallic objects, such as coiling, dental implants/filling and hip/shoulder prostheses. These highly attenuating metallic objects give rise to severe photon starvation, beam hardening and scattering, thus leading to adverse artefacts, including streak, star-shape and voids in the reconstructed CT images. The presence of metallic objects in CT imaging commonly leads to severe deterioration of image quality by causing strong bright and dark streak artefacts, not only in the site of the metallic object but also in the surrounding regions. Metal-induced artefacts could skew the CT signal (depending on the size and material of the metallic object) or generate artificial signals, which might adversely impact the interpretation of CT images and hence potentially clinical diagnosis. In addition to the adverse impact of metal-induced artefacts on the visual assessment of CT images, the quantitative accuracy of the CT signal in the vicinity of metallic objects could be affected [1]. The quantitative analysis of CT images in the vicinity of the metallic object revealed large under/overestimation of tissue densities due to the distortion of the CT signal [2].

In addition to diagnostic imaging applications with contrast enhancement of CT in radiology, this imaging modality is also employed in quantitative positron emission tomography (PET) imaging in the framework of CT-based attenuation correction (AC) of the PET data [3]. Any issues or image artefacts affecting CT images would be reflected in the attenuation-corrected PET images, potentially leading to misdiagnosis and/or quantitative errors [4, 5]. Moreover, CT images commonly serve as standard of reference for the evaluation of MRI-guided attenuation correction [6–8] and MRI-only treatment planning in radiation oncology [9].

The presence of metallic objects in PET imaging itself does not cause any signal loss/distortion; however, the incorrect CT values (due to metal artefacts) could lead to inaccurate PET attenuation and scatter corrections. Noticeable over/underestimation of the activity concentration would be observed in the vicinity of metallic objects. Moreover, strong streak artefacts in CT images may distort genuine PET signal and may also cause factitious signals in the PET image (e.g. false abnormalities). When metallic objects in CT-based PET attenuation map cause inaccurate scatter correction, its impact will not be confounded to the site of the metal artefact and would affect the entire PET volume [4]. Metal artefacts are easily detectable through visual inspection of CT images; however, radiotracer uptake bias in PET images affected by incorrect AC maps owing to metal artefacts is not easily recognized without reviewing the attenuation map. Hence, metal artefact correction in PET imaging is as important as in CT imaging.

To address this issue, a number of metal artefact reduction (MAR) approaches have been proposed in the literature [3, 10]. However, none of them was widely adopted as standard of reference, and, as such, MAR still remains one of the major challenges in CT and PET/CT imaging [11].

A state-of-the-art MAR approach based on the prior image is the normalized MAR (NMAR), wherein thresholding-based image/tissue classification is applied on the original/affected or linear interpolation (LI)–corrected image to generate a prior image [12]. This approach tends to extract as much as possible information from unaffected regions in the original or partly corrected images in order to predict/correct/estimate the missing or affected regions in the original image. Iterative image reconstruction approaches tend to suppress metal artefacts through reconstruction of CT images from the unaffected or preliminary corrected projections/bins [13–16]. Despite these efforts, it is challenging to achieve acceptable/satisfactory outcomes for all metal artefacts using a single MAR framework owing to the high variety of sizes, materials, locations and background structures of metal implants. In this light, several attempts have been made to combine two or three MAR methods to devise a hybrid approach [17]. Hybrid approaches have shown superior performance compared to each of the MAR techniques alone [3]. These methods tend to provide a more realistic/accurate modelling of metal artefacts by taking beam hardening and Poisson noise into account [13] or employing a preprocessing step (for instance using other MAR techniques), which results in a case-specific prior knowledge for the problem at hand [17]. Hence, these methods combine the prior knowledge and/or conventional MAR technique with an iterative image reconstruction algorithm fine-tuned for MAR.

Deep learning–based algorithms emerged as promising approaches for solving a variety of image analysis and pattern recognition problems and have been successfully implemented in the context of metal artefact reduction in CT imaging [18–23]. Deep learning–based MAR techniques could be categorized as the 4th type of MAR approaches, wherein the correction for metal-induced beam hardening is applied in either the projection [22] or image domain [19]. MAR methods based on beam hardening correction, such as NMAR, have limited capability in the presence of high attenuating metal implants. Deep learning–based MAR approaches have demonstrated the ability to refine the performance of the NMAR algorithm, either in the projection or image domain, to eliminate the residual errors or new artefacts introduced by NMAR [24].

The aim of this work is to develop and evaluate a deep learning–based MAR to improve CT-based attenuation and scatter correction in PET/CT imaging. To this end, CT images affected by metallic artefacts were processed by the deep learning–based MAR to assess the impact on the resulting PET images. The proposed approaches were also compared to the NMAR, serving as baseline technique for comparison.

## Materials and methods

### Data acquisition

Two clinical databases were utilized in this work, including a simulation dataset used for training and assessment of the deep learning–based MAR approaches and whole-body ^18^F-FDG PET/CT images used for clinical evaluation. The simulation dataset consisted of 80 metal-free whole-body CT images acquired on a Biograph mCT PET/CT scanner (Siemens Healthcare) using the following parameters: effective tube current = 100 mAs, tube voltage = 110–120 kVp, slice thickness = 3 mm, automated tube voltage selection, automated tube current modulation, pitch factor of 1, mean CTDIvol = 5.8 mGy and DLP of 65 mGy·cm (average BMI = 29.5). Eighty patients selected from whole-body CT scans with no metal artefact (metal artefact–free) were included in the simulation dataset for the training of the network.

The PET/CT dataset used for clinical evaluation consists of 30 patients (mean age ± SD = 60 ± 8 years and mean weight ± SD = 70 ± 8 kg) presenting with substantial dental fillings (24), hip (3) and shoulder joint prostheses (3). For clinical evaluation, 30 patients presenting with substantial dental fillings, hip and shoulder joint prostheses were selected from whole-body PET/CT scans. Whole-body PET/CT scans were performed on the same Biograph mCT scanner (using the same CT acquisition protocol) about 1 h post-injection of 250±45 MBq of ^18^F-FDG for an average acquisition time of 25±5 min. The study protocol was approved by Geneva Cantonal Research ethics committee.

### Metal artefact simulation

The training of the deep learning–based MAR approaches involved the use of 80 metal-free CT images where metals were artificially inserted to create a metal artefact–affected dataset with known ground truth. These data contained metal-free, metal-inserted (ground truth images) and metal-affected CT images. Metal-free images are the original CT images whereas the metal-inserted images were generated by inserting metallic implants in the metal-free images. These metallic implants included dental fillings, spine fixation screws and hip and shoulder joint prostheses with linear attenuation coefficients of iron, copper, gold and titanium. The algorithm proposed in [19] was employed to simulate metallic artefacts in CT images (metal-affected images), wherein the metal-inserted images were segmented into soft tissue, bone and metal using a weighted thresholding-based approach [25] followed by assignment of the corresponding attenuation coefficients. Subsequently, the metal-affected CT images were reconstructed through a combination of forward projection and filtered backprojection approach. The adopted metal artefact simulation approach is briefly described below [19]. Further details about the simulation process are provided in Supplemental materials.

The simulation dataset consisted of 80 clinical CT images (45 men and 35 women, age 51 ± 12 years) with no metal implants. On this dataset, 8 different metal shapes were simulated using 4 different materials (namely iron, gold, titanium and copper) in the different regions of the body. For each case (shape and material), 25 metal artefact simulations were conducted resulting in 25 (realizations) × 8 (shapes) × 4 (materials) metal artefact–affected images/samples. Given the 800 simulated cases, 660 were employed for the training and 140 for the evaluation of the different methods, equally sampled from the different metal shapes, materials and anatomical regions. Mass attenuation coefficients of the metal inserts, bone and soft tissue were obtained from the XCOM photon cross section library [26]. The metal-free and metal-inserted images underwent a polychromatic Radon transform (following the algorithm described in [19]) and were reconstructed using a filtered backprojection (FBP) algorithm to generate standard of reference images (for evaluation of MAR techniques) and the metal-affected images, respectively. An X-ray source with 120 kVp tube potential and equi-angular fan-beam geometry was simulated. One thousand angular projections over 920 detector bins were calculated for each CT slice.

### Metal artefact reduction strategies

#### Normalized metal artefact correction

The normalized MAR (NMAR) approach relies on prior knowledge obtained from a threshold-based image classification of the metal-affected or LI corrected image. In the first step, the metal implant is segmented from the input image followed by forward projection and the identification of the metal trace in the projection space. Prior knowledge is used to normalize the metal-affected data in the projection space before applying interpolation to the trace of the metal implant [12].

The NMAR approach was applied on both real (clinical) and simulated metal-artefacted images, providing a baseline for evaluation of the deep learning–based MAR approaches. Moreover, the outcome of the NMAR approach was employed as prior knowledge or auxiliary image to be fed into the deep learning model.

#### Deep learning–based MAR in the image domain

**Fig. 1:**
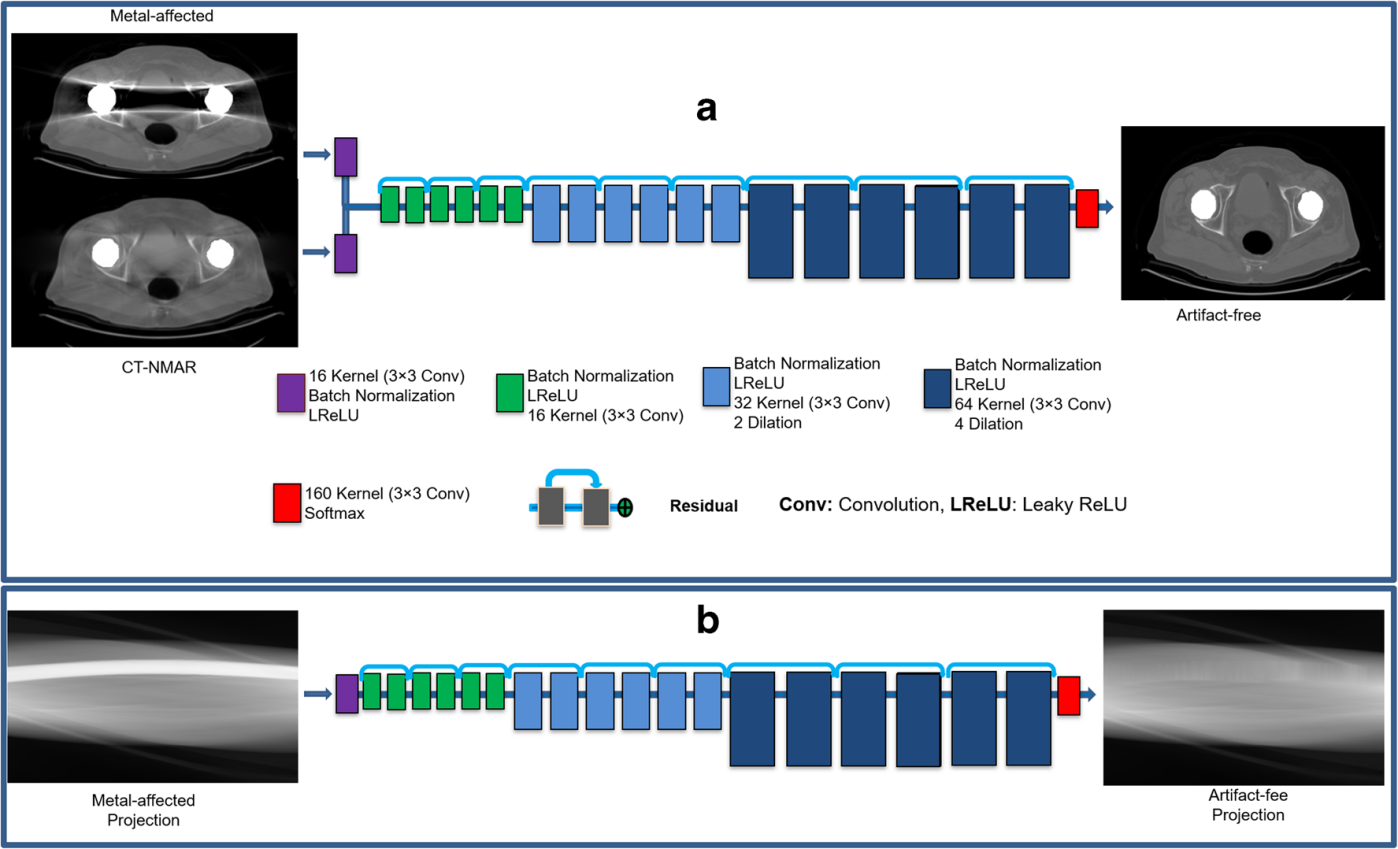
Architecture of the HighResNet model used for (**a**) deep learning–based metal artefact reduction in the image domain (DLI-MAR) and (**b**) deep learning–based metal artefact reduction in the projection domain (DLP-MAR)

Deep learning–based MAR can be implemented in either the projection or image domains (or any combination of them). MAR in the image domain is considered as an end-to-end image transformation or regression from metal-affected to artefact-corrected (or artefact-free) CT images. To implement MAR in the image domain, three different strategies were explored: (i) the input image to the deep learning model is restricted to the metal-affected image to estimate the artefact-corrected CT image, (ii) the input image is the metal artefact–corrected CT image using the NMAR technique (CT-NMAR), (iii) the input to the deep learning model consist of both metal-affected and CT-NMAR images fed in two different channels (Fig. 1a). Visual inspection and preliminary quantitative analysis revealed the superior performance of the third scenario. Hence, in the rest of this work, we focus only on this approach.

Two-hundred metal-free, simulated metal-inserted and metal-affected volumetric CT images were employed to train the deep learning model in the image domain. To this end, a dilated convolutional neural network with residual connections (HighResNet) [27] was adopted and configured in the open-source NiftyNet deep learning platform [28]. Overall, 10,000 2D simulated metal-affected and metal-inserted CT images were used for the training whereas 2000 2D CT images were used for evaluation of the model. The model consisted of two entry channels of 400 × 400 voxels taking metal-affected and CT-NMAR images as input and one output channel of 400 × 400 voxels. The HighResNet network is composed of 20 residual convolutional layers regulated by 3 × 3 × 3 kernels dilated by factors two and four at the different layers (Fig. 1a). To train the model, an L2 loss function was adopted using the Adam optimization and learning rate from 0.06 to 0.01 following the procedure recommended in [29].

#### Deep learning–based MAR in the projection domain

**Fig. 2:**
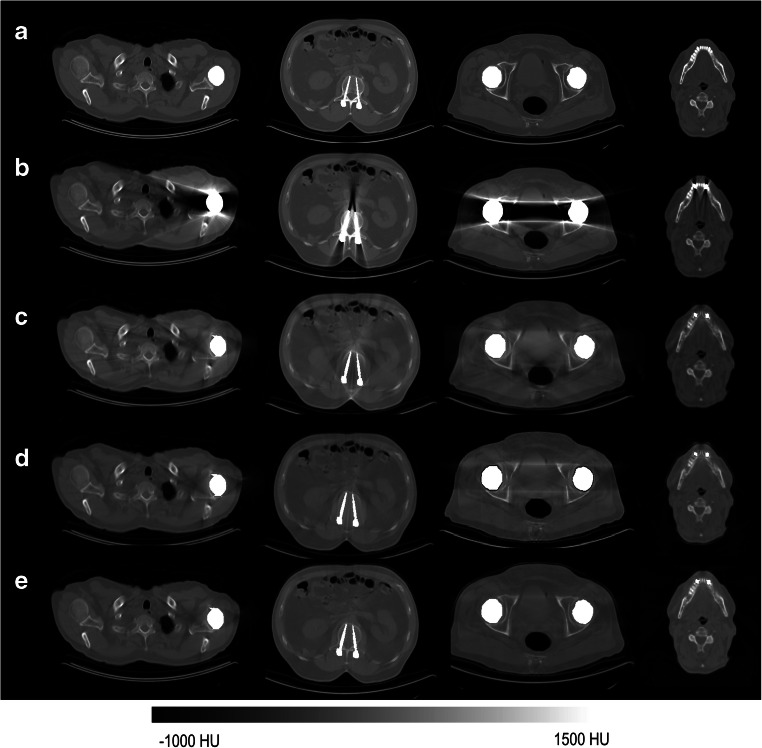
Representative examples of metal artefacts simulation and correction showing **a** CT images after inserting metallic implants (standard of reference). **b** Metal-affected CT images. CT images after applying MAR using (**c**) NMAR, (**d**) deep learning algorithm operating in the projection domain (DLP-MAR) and (**e**) deep learning algorithm operating in the image domain (DLI-MAR)

To implement the deep learning–based MAR in the projection domain, the same three strategies explored in the image domain were adopted, except that image-to-image regression was carried on 2D CT projection data after applying the Radon transform. Contrary to the image domain, none of the three strategies exhibited superior performance. However, the first strategy, wherein the input to the deep learning model is the metal-affected projections to generate the artefact-free projections (Fig. 2b), was selected owing to its higher robustness and computational efficiency. The same deep learning model and training procedure described in the previous section were used to implement MAR in the projection domain. The resulting artefact-free projections were reconstructed using the FBP algorithm with 1000 angular projections over 920 detector bins.

### MAR in PET/CT imaging

To evaluate the impact of MAR in whole-body PET/CT imaging, a clinical database consisting of 30 patients presenting with severe metal artefacts in CT images was collected. The metal implants are dental fillings (24), hips (3) and shoulder joint prostheses (3). The raw PET data were corrected for attenuation using the original CT images (before MAR), CT images corrected by NMAR algorithm and CT images corrected by the deep learning approaches. The quality and quantitative analysis of PET images corrected for attenuation using CT images before and after MAR was assessed to put into perspective the potential impact of MAR in PET imaging.

The PET raw data were reconstructed using 3D ordinary Poisson-ordered subset expectation maximization (OP-OSEM) algorithm with 2 iterations and 21 subsets in 400 × 400 matrix with a voxel size of 2 × 2 × 3 mm. CT-based scatter correction, time-of-flight (TOF) information and point spread function (PSF) modelling were considered during PET image reconstruction using the offline e7 tool (Siemens Healthcare).

### Evaluation strategy

Evaluation of MAR techniques was carried out on CT and PET images corrected for attenuation using CT images before and after applying MAR. For quantitative evaluation of CT images, peak signal-to-noise ratio (PSNR), root mean square error (RMSE) and structural similarity (SSIM) index were calculated before and after MAR using Eqs. (1)–(3), respectively.

1 $$ PSNR(dB)=10{\log}_{10}\left(\frac{Pk^2}{MSE}\right) $$

2 $$ RMSE=\sqrt{\frac{1}{vxl}\sum \limits_{v=1}^{vxl}{\left({CT}_{\mathrm{MAR}}(i)-{CT}_{\mathrm{ref}}(i)\right)}^2} $$

3 $$ SSIM=\frac{\left(2{Ave}_{\mathrm{ref}}{Ave}_{\mathrm{MAR}}+{k}_1\right)\left(2{\delta}_{\mathrm{ref},\mathrm{MAR}}+{k}_2\right)}{\left({Ave}_{\mathrm{ref}}^2+{Ave}_{\mathrm{MAR}}^2+{k}_1\right)\left({\delta}_{\mathrm{ref}}^2+{\delta}_{\mathrm{MAR}}^2+{k}_2\right)} $$

In Eq. (1), *Pk* indicates the maximum intensity of either *CT*_ref_ or *CT*_MAR_ whereas *MSE* denotes the mean squared error between reference CT images (metal-inserted) and CT images after MAR. In Eq. (3), *Ave*_ref_ and *Ave*_MAR_ are the mean values of *CT*_ref_ and *CT*_MAR_ images, respectively. *δ*_ref_ and *δ*_MAR_ stand for the variances and *δ*_ref,MAR_ denotes the covariance of *CT*_ref_ and *CT*_MAR_ images. The constant parameters (*k*_1_ = 0.01 and *k*_2_ = 0.02) were sought to avoid division by very small values. The metal-inserted CT images with no artefacts (generated by the simulation) were considered as reference.

Thirty clinical whole-body PET/CT images with noticeable metal artefacts were examined before and after applying MAR to CT images. The evaluation focused on artefacts induced on PET images by metal artefact present in the attenuation maps as well as PET quantification bias in the vicinity of the metallic implants before and after MAR. Moreover, the impact of MAR on scatter correction and consequently on PET quantification was assessed using whole-body ^18^F-FDG PET/CT studies. To this end, standardized uptake values (SUVs) were calculated within volumes of interest (VOIs) drawn on the affected regions before and after applying MAR using the different techniques. The VOIs were defined in such a way to cover a sufficiently large volume encompassing the entire metallic object, the surrounding affected/disturbed tissues and the residues of the metal artefacts. Larger VOIs were drawn for hip and shoulder joint prostheses with a maximum size of 950 cm^3^ whereas smaller VOIs were drawn for dental fillings with a minimum size of 55 cm^3^.

The significance of the differences between the different MAR techniques (quantitative metrics) was assessed using the paired *t* test method. *P* values smaller than 0.05 were regarded to reflect statistical significance.

## Results

In the first part of the “Results” section, we report the evaluation of MAR techniques solely on CT images using the simulated metal artefacts dataset. Subsequently, the impact of metal artefacts (in CT images) and the different MAR techniques are assessed on the corresponding attenuation-corrected PET images.

Figure 2 (complete set of images is presented in Supplemental Figure 1) illustrates four examples of metal artefacts and the outcome of different MAR techniques from the simulation dataset. The metal-inserted images (artefact-free CT images inserted with metal implants) are regarded as standard of reference. Deep learning–based MAR in the image (DLI-MAR) and projection (DLP-MAR) domains were assessed along with NMAR techniques, which provides a yardstick for comparison. Visual inspection revealed the superior performance DLI-MAR compared to NMAR and DLP-MAR techniques.

Table 1 summarizes the performance metrics of the different MAR techniques on the simulation dataset. In agreement with visual inspection (Fig. 2), quantitative evaluation of the MAR techniques demonstrated the superior performance of DLI-MAR techniques. The *p* values in Table 1 were calculated between the results obtained from DLI-MAR and DLP-MAR (reported in the same table) techniques to examine the statistical significance of the differences between these two techniques.

**Table 1:** RMSE, SSIM and PSNR calculated on metal-affected (prior to MAR) CT images and after application of NMAR, deep learning–based MAR in the projection domain (DLP-MAR) and deep learning–based in the image domain (DLI-MAR) techniques on the simulation dataset. *p* values were calculated between the results obtained from DLP-MAR and DLI-MAR techniques

	RMSE	SSIM	PSNR
Metal-affected	169 ± 84 HU	0.58 ± 0.8	25.1 ± 2.5
NMAR	47 ± 33 HU	0.93 ± 0.3	35.8 ± 2.1
DLP-MAR	33 ± 21 HU	0.94 ± 0.2	37.6 ± 2.0
DLI-MAR	29 ± 17 HU	0.95 ± 0.2	38.2 ± 2.0
*p* value	< 0.02	< 0.05	< 0.05

The validation dataset consisted of 140 samples of simulated metal artefacts generated from the different metal shapes, materials and anatomical regions. A separate analysis of the results associated with a specific metal shape, material or anatomical region revealed no significant differences and variations among them. Hence, we concluded that the performance of the evaluated techniques is not correlated/linked to any specific anatomical region, metal shape or material. The average values over the entire validation cases are reported for each technique in Table 1. A similar observation was made in the clinical study wherein the different techniques performed similarly in the different regions of the body.

Although the evaluation of MAR techniques on CT images demonstrated the superior performance of DLI-MAR over DLP-MAR, there was no proof of statistically significant differences when the resulting CT images were used for PET attenuation correction. Therefore, only the results obtained from NMAR and DLI-MAR methods were presented when assessing the impact of MAR techniques on PET images.

**Fig. 3:**
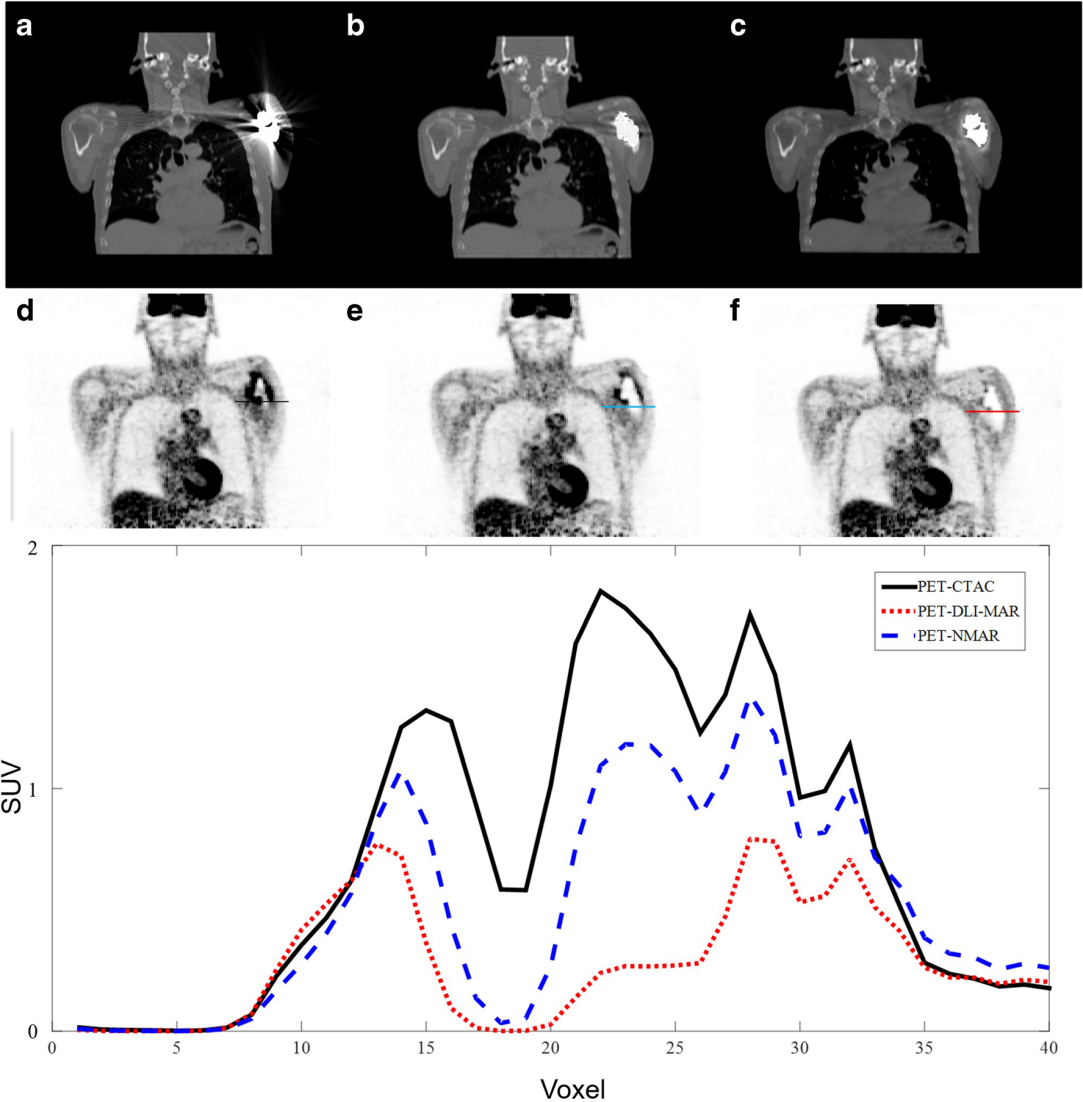
Representative example of image artefacts apparent on PET images from shoulder joint prosthesis in CT images. Original CT image before correction (**a**) and after MAR using NMAR (**b**) and DLI-MAR (**c**) methods. The corresponding attenuation-corrected PET images are displayed in **d**, **e** and **f**, respectively. Horizontal profiles drawn on the shoulder area of PET images show the impact of metal artefacts and their reduction on the resulting PET signals

**Fig. 4:**
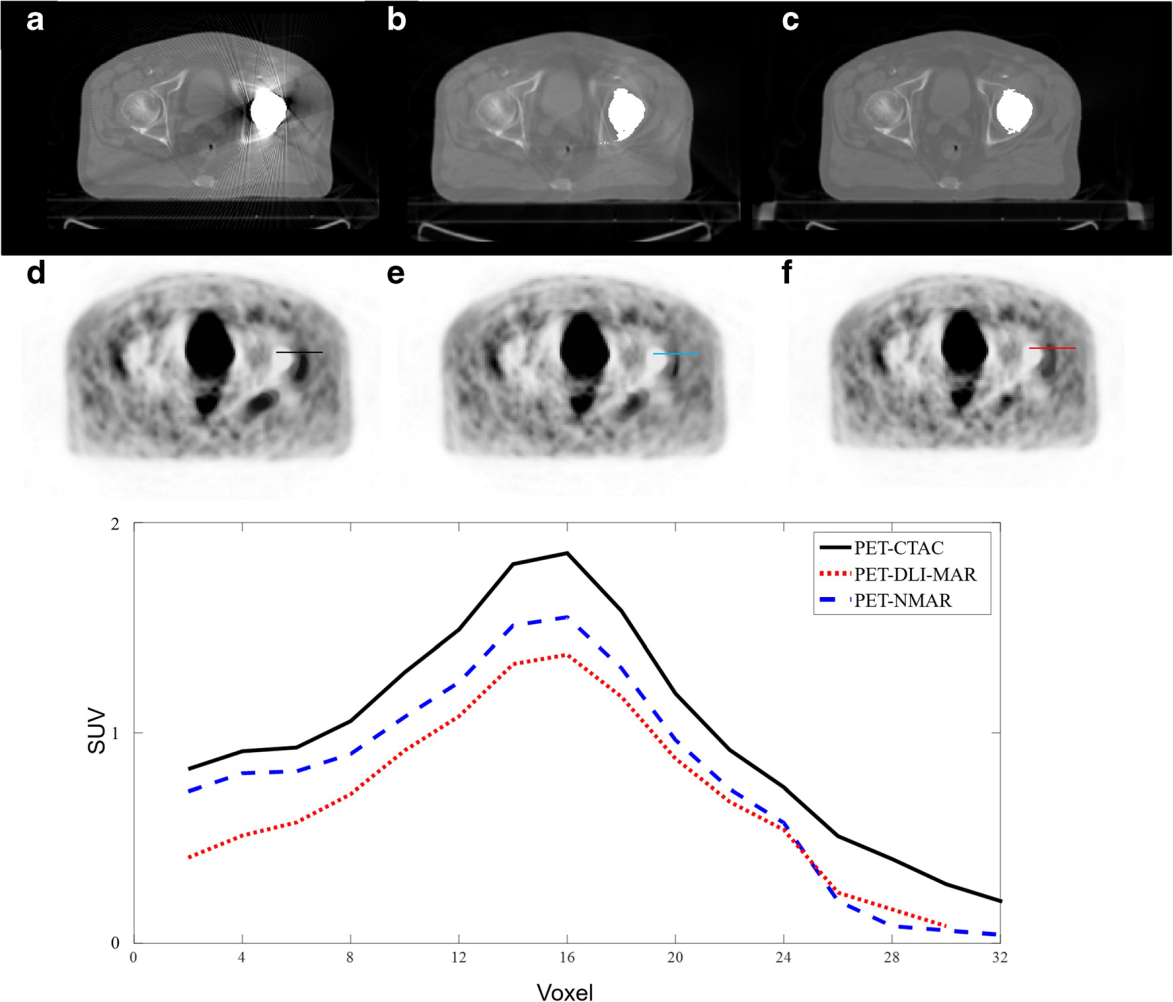
Representative example of image artefacts apparent in the PET image from the hip prosthesis in CT images. Original CT image before correction (**a**) and after MAR using NMAR (**b**) and DLI-MAR (**c**) methods. The corresponding attenuation-corrected PET images are displayed in **d**, **e** and **f**, respectively. Horizontal profiles drawn on the pelvis area of PET images show the impact of metal artefacts on PET signals

Figures 3 and 4 present metal artefacts in CT images due to shoulder joint and hip prostheses, which gave rise to image artefacts in the corresponding PET images. The image artefacts are clearly visible around the site of metallic implants. MAR techniques, particularly DLI-MAR, eliminated noticeably the streak and dark bands from CT images, consequently reducing the artefacts around the site of the implants. The horizontal profiles drawn on PET images illustrate clearly the degree of PET signal alteration before MAR and recovery by the MAR techniques.

**Fig. 5:**
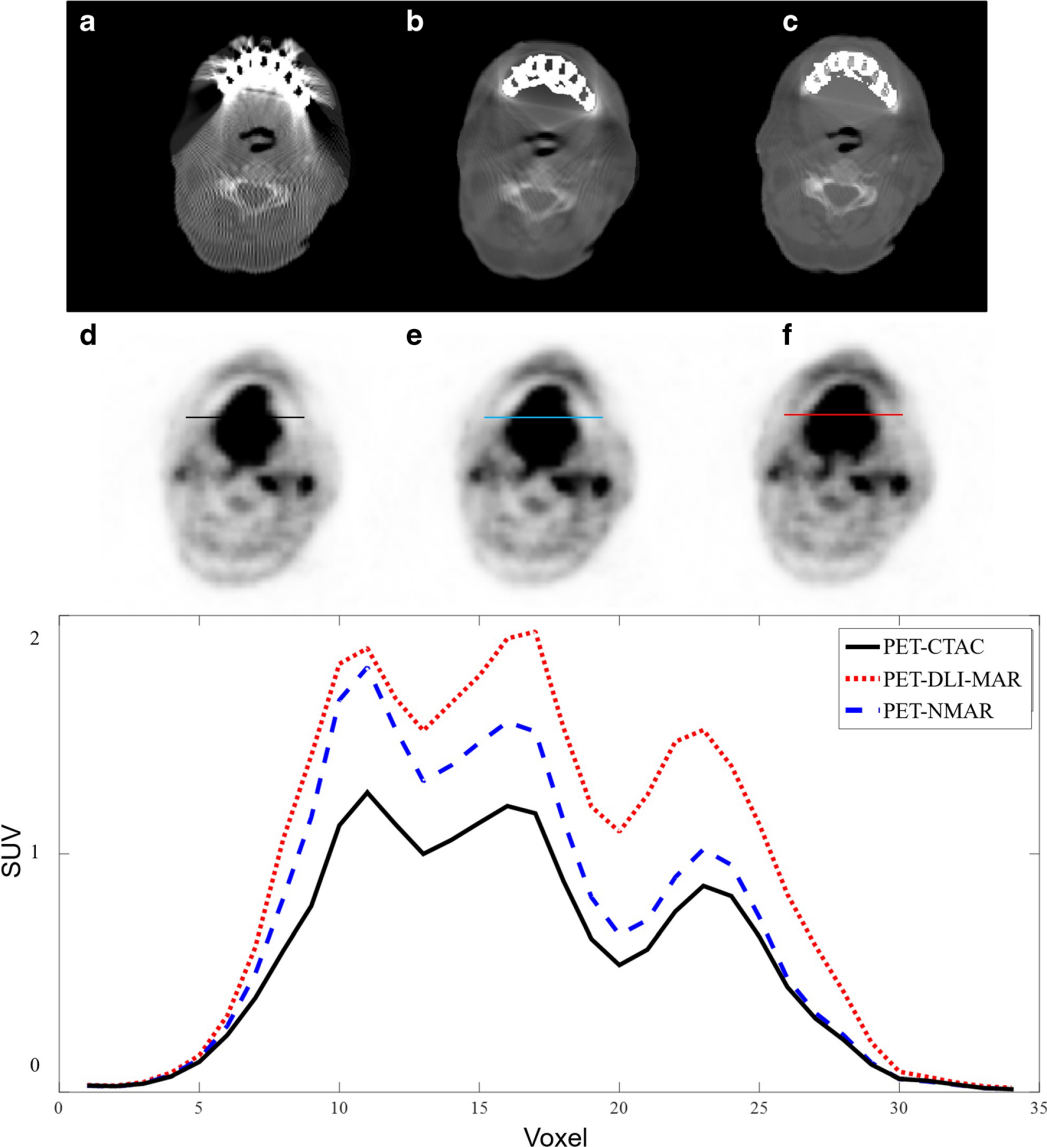
Example of scatter correction overestimation due to metallic artefacts. Original CT image before correction (**a**) and after MAR using NMAR (**b**) and DLI-MAR (**c**) methods. The corresponding attenuation-corrected PET images are displayed in **d**, **e** and **f**, respectively. Horizontal profiles drawn on PET images compare the PET signals before and after application of MAR methods

**Fig. 6:**
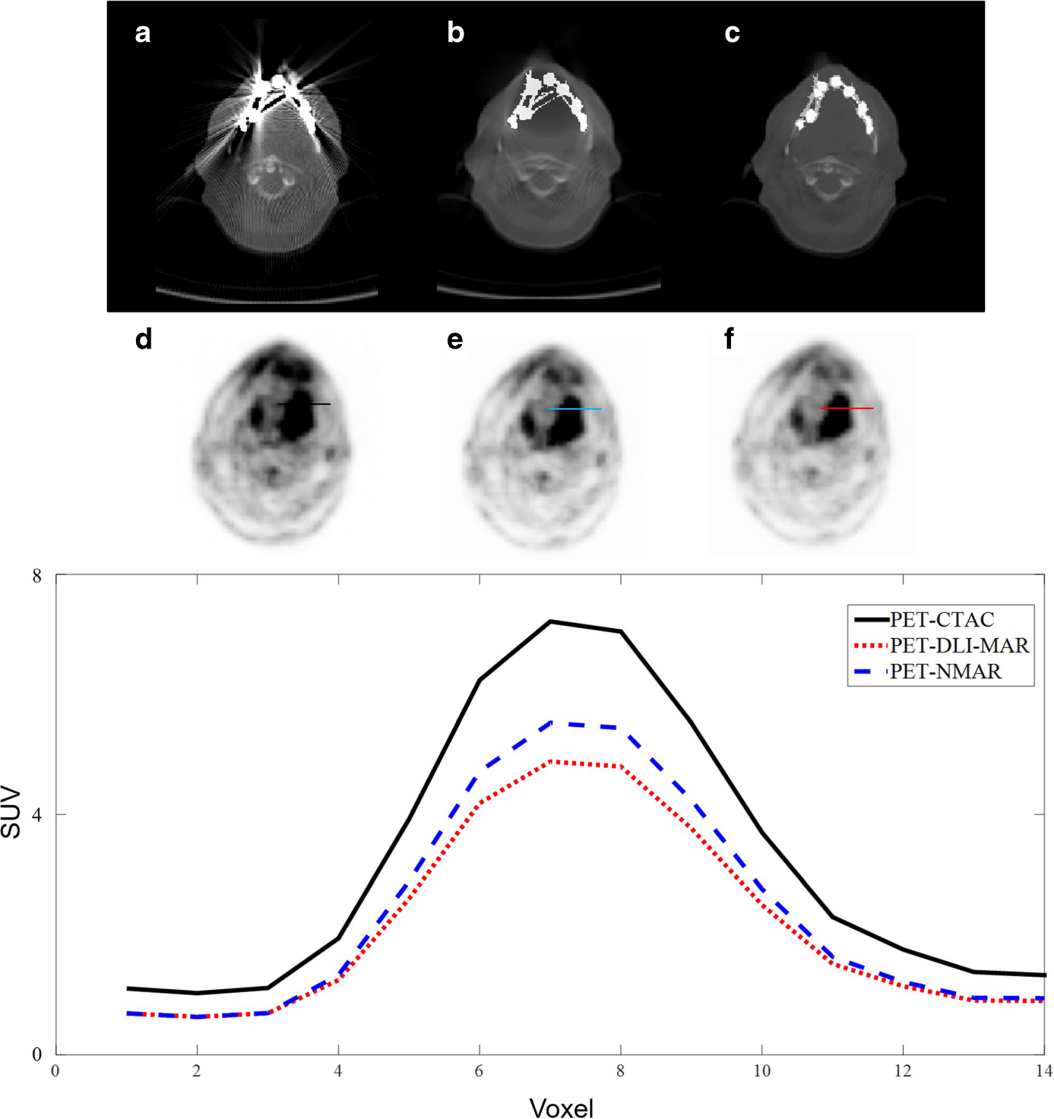
Overestimation of tracer uptake (SUV) in a malignant lesion due to metallic artefacts. Original CT image before correction (**a**) and after MAR using NMAR (**b**) and DLI-MAR (**c**) methods. The corresponding attenuation-corrected PET images are displayed in **d**, **e** and **f**, respectively. Horizontal profiles drawn on the lesion on PET images compare the PET signals before and after application of MAR methods

Figures 5 and 6 depict two examples of impaired scatter estimation during PET image reconstruction owing to the presence of metallic artefacts in CT images. In Fig. 5, metal artefacts led to underestimation of the head contour due to the weak signals at the boundary of the head, which resulted in overestimation of the scatter fraction when using model-based scatter correction [30]. The scatter fraction for this bed position for PET-CTAC was 58% while scatter fractions of 33% and 24% were observed when using PET-NMAR and PET-DLI-MAR, respectively. Horizontal profiles drawn on PET images show the differences between PET images before and after applying the different MAR techniques.

Supplemental Figure 2 depicts an example wherein metal artefacts in CT images led to significant underestimation of scatter fraction during PET/CT image reconstruction. The scatter fraction in PET-CTAC was 11%, while scatter fractions of 19% and 25% were observed in PET-NMAR and PET-DLI-MAR images, respectively.

Figure 6 depicts an example wherein the lesion near the site of metal artefact was significantly overestimated in the PET-CTAC image. Application of MAR techniques on the CT image or attenuation map significantly reduced the seemingly artificial high SUV of the lesion.

Table 2 summarizes the quantitative impact of MAR techniques on PET images. Thirty VOIs were drawn at the site of metal artefacts on both clinical and simulation datasets. For the simulation dataset, the mean SUV bias (percentage of SUV under-/overestimation after MAR averaged across all patients) was measured within the VOIs considering the metal-inserted images (CT images with metal implant without artefacts) as standard of reference attenuation maps. For the clinical dataset, the bias (or change in tracer uptake) was calculated against the PET-CTAC before applying the MAR techniques owing to the lack of ground truth attenuation map. Overall, the simulation studies demonstrated that metallic artefacts in CT images led to significant overestimation of the SUV in the proximity of the metallic implant, while MAR techniques, in particular DLI-MAR, were able to reduce the adverse impact of the metallic artefacts on PET images. The large negative bias observed in the clinical studies confirmed the observations in the simulation dataset.

**Table 2:** Mean SUV bias within the 30 VOIs drawn at the site of metallic artefacts on the clinical and simulation studies. For the simulation dataset, the metal-inserted CT images (artefact-free) were considered as reference. For the clinical dataset, the SUV bias for MAR techniques was calculated against PET-CTAC images before applying MAR owing to the absence of ground truth. *p* values calculated between the results obtained from NMAR and DLI-MAR techniques are also shown

	PET-CTAC	PET-NMAR	PET-DLI-MAR	*p* value
Clinical data	-	−5.8 ± 4%	−7.6 ± 5%	< 0.05
Simulation data	10.5 ± 6%	3.2 ± 5%	1.3 ± 3%	< 0.01

## Discussion

Different strategies for deep learning–based metal artefact reduction (MAR) of CT images were explored either in the image or projection domains [18–22]. The evaluation of CT images after MAR demonstrated the superior performance of the deep learning–based MAR in the image domain (DLI-MAR) when additional information (prior knowledge) in the form of CT images corrected by the normalized MAR approach was also fed to the network (Fig. 1a). Similar observations were reported in other studies [19] wherein the deep learning model configured in the image domain performed better when the output of other MAR methods was employed as prior knowledge (additional input channels). Though deep learning–based MAR in the projection domain (DLP-MAR) outperformed NMAR approach, it lagged behind the DLI-MAR approach. Upgrading the DLP-MAR approach (Fig. 1b) through assigning an extra input channel to take the prior knowledge in the form of CT projections corrected by the NMAR approach did not improve the overall performance of the DLP-MAR approach. Since the anatomical information present in the image domain is more discernible and extractable for deep learning models, DLI-MAR resulted in more accurate estimation of the artefact-free CT images.

The major focus of this study was on the impact of MAR techniques on CT-based attenuation correction in PET to investigate the quantitative bias and potential image artefacts. Nevertheless, the evaluation of MAR techniques was carried out on CT images alone using the SSIM and PSNR metrics, which reflect the perceived quality of corrected CT images and an approximation to human perception, respectively. These parameters reveal the quality or resemblance of the predicted CT images to the originals (ground truth), representing the quality of CT images for visual inspection. Moreover, the RMSE reflects the accuracy of the predicted CT numbers, which is relevant for quantitative analysis of CT images and CT-based attenuation correction in PET.

Despite the superior performance of DLI-MAR compared to DLP-MAR when the evaluation is performed on CT images, no significant differences were observed on the quantification of PET images when the output of these approaches were utilized for PET attenuation correction. One possible explanation of this observation is that (i) for PET AC, CT images are commonly downsampled to the resolution of the PET images (e.g. from 0.97 mm CT resolution to 4 mm PET resolution) and smoothed using a Gaussian filter. Thus, many nuances and small defects would disappear in the generated AC maps. (ii) The sensitivity of PET image reconstruction to attenuation correction is not up to the level where any defects in the AC maps are fully reflected on PET images [31–33].

The assessment of deep learning–based MAR approaches in the literature focused mostly on evaluating the impact on the quality of CT images [34]. Metal artefacts in CT-based attenuation maps give rise to skewed information about the attenuating medium in regions far away from the sites of metallic implants. Metal-induced streak artefacts commonly cause overestimation of attenuation factors, consequently leading to inaccurate quantification and/or image artefact in PET images. Metal artefacts in PET AC maps lead to image artefacts and/or overestimation of tracer uptake in PET images (Figs. 3, 4 and 6). SUV changes of up to 11% have been reported in comparative studies assessing the impact of different MAR techniques on PET quantification [35, 36]. However, in very few cases, metal artefacts resulted in the overestimation or dilation of the body contour which misled the scatter correction algorithm, resulting in the underestimation of the scatter fraction. The single-scatter simulation algorithm, implemented on the Biograph mCT PET/CT scanner, takes into account only single Compton scatterings and relies on a tail fitting approach to estimate the total scatter faction [30, 37]. As a result of this tail fitting, overestimation of the body contour would lead to underestimation of the scatter fraction (Fig. 5) and vice versa (Supplemental Figure 2). It should be noted that these signal differences are not only due to scatter correction (or scatter fraction) since CT images or attenuation maps are also different after applying the MAR techniques. Scatter correction in PET imaging is not very sensitive to variations of the voxels intensity in the attenuation map [38, 39]. However, estimation of the scatter fraction through the tail fitting approach is highly sensitive to the boundary of the attenuation map to differentiate the genuine uptake signals from scattered events.

Although recent studies have demonstrated the superior performance of deep learning methods [20], a hybrid method would be able to provide more dependable MAR solutions having the merits of maximal signal recovery and low likelihood of introducing new artefacts. The deep learning methods, which incorporate/rely on an analytic model–based technique as prior knowledge, would be able to offer an optimal solution for MAR, enabling efficient recovery of underlying structures and tissue densities. The deep learning–based MAR, as a decision support tool, would be able to prevent gross errors and/or misdiagnosis in PET/CT imaging.

Due to the lack of ground truth in clinical studies, accurate quantitative analysis of MAR approaches is very challenging if not possible at all [40]. Nevertheless, the simulation studies demonstrated that metal artefacts in CT images lead to overall overestimation of local tracer uptake in PET images (Table 2). The results of the clinical studies confirmed the observations made on the simulation dataset wherein PET-CTAC images before MAR exhibited higher local activity concentration compared to either PET-NMAR or PET-DLI-MAR images.

One of the major limitations of deep learning–based solutions in general and the MAR algorithm proposed in this work in particular is the dependency of performance on the training dataset. Metal artefacts could occur in various anatomical sites. Hence, developing a comprehensive deep learning–based MAR model requires the creation of a large training dataset including potential diversity of metal shapes, materials and anatomical variability. This study tended to focus on the major/most probable metal artefact cases normally occur due to dental implants, hip, shoulder and spine fixations. However, a comprehensive/versatile model should involve all metal artefact manifestations. Moreover, the proposed MAR model is trained and optimized for a specific CT scanner and acquisition protocol, which might perform sub-optimally when using CT images with different noise levels or image characteristics. Transfer learning could be an efficient solution to this issue wherein fine-tuning of the developed model could be carried out using a training dataset from a different CT scanner or acquisition protocol. Last but not least, the proposed deep learning–based MAR technique requires slight intervention by the user to define/window the site of the metal artefact to feed the deep learning–based MAR model with the extracted sub-volume of the image. This may be considered as a practical limitation of this model compared to fully automated MAR algorithms.

It can be concluded that we evaluated the potential of deep learning–based metal artefact correction in PET/CT imaging. Deep learning–based MAR in the image domain (DLI-MAR) outperformed its counterpart implemented in the projection (DLP-MAR) domain. Metal implants gave rise to image artefacts, quantitative bias, and under- or overestimation of scatter correction in PET imaging. The DLI-MAR approach was capable of minimizing the adverse impact of metal artefacts on whole-body PET images through generating accurate attenuation maps from corrupted CT images.

## Supplementary Information

ESM 1 (DOCX 4209 kb)

## Abbreviations

^18^F-FDG: ^18^F-Fluorodeoxyglucose
CNNs: Convolutional neural networks
CT: Computed tomography
DLI-MAR: Deep learning–based MAR in the image space
DLP-MAR: Deep learning–based MAR in the projection space
LI: Linear Interpolation
MAR: Metal artefact correction
NMAR: Normalized metal artefact correction
PET: Positron emission tomography
PET/CT: Positron emission tomography/computed tomography
SUV: Standardized uptake value
TOF: Time of flight
VOI: Volume of interest
WB: Whole body
