# Supplementary material for: Deep learning–based metal artefact reduction in PET/CT imaging
Source: Eur Radiol. 2021 Feb 10;31(8):6384–96. doi: 10.1007/s00330-021-07709-z (PMC8270868; doi:10.1007/s00330-021-07709-z)
Supplement: Supplementary file 1 — (DOCX 4209 kb) [file 330_2021_7709_MOESM1_ESM.docx]

**Supplemental Material**

**Metal artefact reduction techniques**

Overall, MAR approaches can be classified into two categories: implicit and explicit [1]. Implicit methods commonly manipulate the acquisition parameters prior to the scanning procedures [2]. Explicit methods are divided into: (i) correction methods for physical effects, (ii) interpolation and non-interpolation approaches in the projection domain, (iii) iterative image reconstruction algorithms, (iv) hybrid approaches which combine any of the above mentioned techniques [3], and (v) image-based approaches wherein the affected pixels are replaced with constant values instead of manipulating raw CT data [4]. Correction for physical effects, such as beam hardening [5] and photon starvation [6] could directly reduce artifacts in CT images. However, the signals loss/distortion are so severe due to the presence of highly attenuating metallic implants that these corrections would not achieve satisfactory results. In the projection domain, metal-affected bins could be assumed as missing signal/information, wherein linear interpolation (LI) is broadly employed to approximate the missing information through exploiting the neighboring unaffected projection bins [7]. The LI approach is not able to fully recover the missing signals and may introduce new artifacts or distortion of structures in the proximity of the metal implants [8]. This issue was partly compensated through employing a priori information in the forward projection, leading to a more accurate substitute for missing signals [9].

**Metal artefact simulation**

To ensure a realistic metal-artifact simulation, instead of using phantom imaging, metal implants, representing typical shape and size of prostheses and dental implants encountered in clinical studies were manually segmented from patients’ CT images. Given the binary masks of metallic objects, metal-inserted CT images were generated through superimposition of the metallic objects on the metal-free CT images in different locations, orientations, sizes (slightly larger or smaller) and materials. Then, the pixel values were converted from Hounsfield Units (HUs) to linear attenuation coefficients. To generate polychromatic projection data, the metal-inserted images were segmented into water, bone and metallic implants using a thresholding-based classification approach [10]. Pixels with values below 100 HUs were considered as soft-tissue (or water-equivalent) whereas pixel values above 1500 HUs were considered as bone. Pixels with values between these two threshold levels were linearly weighted to create a mixture of water and bone. For metal artifact simulation, binary masks of metallic objects were placed into corresponding/possible anatomical locations to generate metal-inserted images. The most likely and significant metal implants, such as dental fillings, spine fixation screws, hip and shoulder joint prostheses were simulated followed by assignment of linear attenuation coefficients of iron, copper, gold and titanium.

**Deep learning algorithm**

A unique capability of deep learning approaches is image-to-image translation enabling the prediction of different representations of the input images or inter-modality translation. This capability can be used to estimate the original/uncorrupted/corrected version of the input images affected by certain degrading factors. Metal artifact reduction (MAR) in CT images is a suitable target for deep learning-based solutions wherein the corrected images could be predicted from the corrupted input images in an end-to-end fashion. To this end, the deep learning network would extract the underlying image features from the input data, based on which an optimized solution is derived to synthesize the original/uncorrupted images. The ability of the deep learning approach to identify/decode the discriminative/underlying features from the input data, which provide sufficient/adequate information to estimate the original/uncorrupted images, plays a key role in the development of an optimal MAR solution. Besides, the network should be able to construct an efficient model to generate/predict the corrected images based on the extracted features. These two cores of the deep learning network should be jointly trained/optimized to reach an overall dependable solution. The HighResNet network selected for this task offers a high-resolution feature extraction scheme (to develop a rich feature map) and a powerful image formation ability which is ideal for application in MAR. The HighResNet network relies on several residual blocks to achieve an efficient training process without increasing the computational cost. Residual or shortcut connections are intended to skip certain layers in a network to form a fluid/uniform propagation of the information across the entire layers (avoiding gradient vanishing). The residual connections would transmit signals (concerning the extracted features or estimated errors or feedback from the ground truth) forward and backward within the blocks. In this regard, a network with n residual blocks would create 2n different paths creating an effective large receptive field.

This network consists of 20 convolutional layers, where residual connections are employed to link every two convolutional layers. Each convolutional layer is also linked to an element-wise rectified linear unit (ReLU) and a batch normalization (BN) layer. In the initial layers, 3×3×3 filters/kernels are employed, which guarantees high-resolution feature extraction from the input image. The layers residing deeper in the network employ dilated kernel with factors of two and four to create larger receptive fields. The output of the network is a fully connected softmax layer having the same matrix size as the input image.


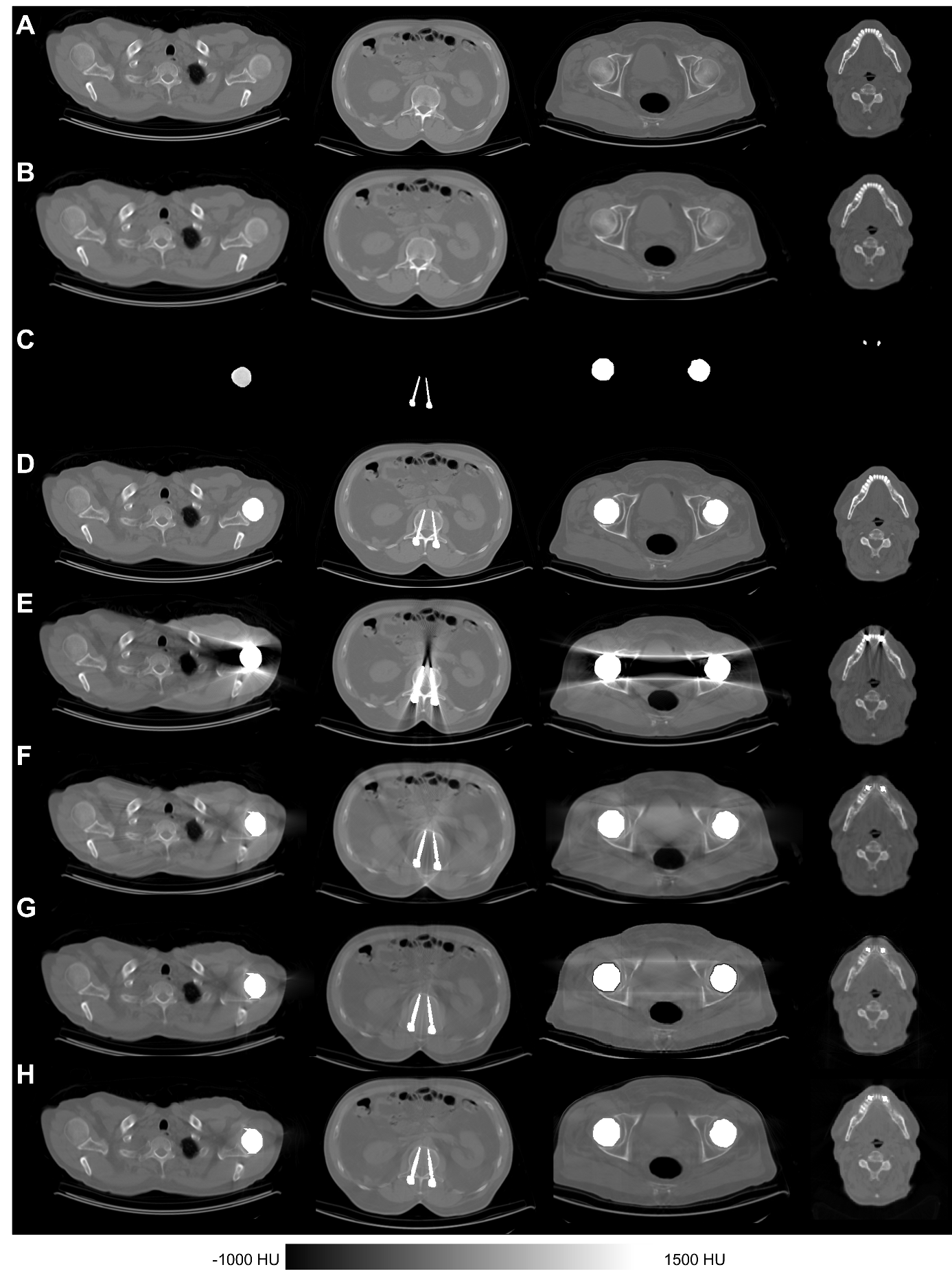


**Supplemental Figure 1**. Representative examples of metal artifacts simulation and correction. (A) Original metal-artifact free CT images. (B) Original CT images after forward-projection and FBP reconstruction. (C) Metallic implants. (D) CT images after inserting metallic implants (standard of reference). (E) Metal-affected images. CT images after applying MAR using (F) NMAR, (G) deep learning algorithm operating in the projection domain (DLP-MAR), and (H) deep learning algorithm operating in the image domain (DLI-MAR).

**Supplemental Figure 2.** Example of scatter correction underestimation due to metallic artifacts. Original CT image before correction (A) and after MAR using NMAR (B) and DLI-MAR (C) methods. The corresponding attenuation corrected PET images are displayed in (D), (E) and (F), respectively. Horizontal profiles drawn on PET images compare PET signals before and after application of MAR methods.

**References**

1 Abdoli M, Dierckx RA, Zaidi H (2012) Metal artifact reduction strategies for improved attenuation correction in hybrid PET/CT imaging. Med Phys 39:3343-3360

2 Coolens C, Childs PJ (2003) Calibration of CT Hounsfield units for radiotherapy treatment planning of patients with metallic hip prostheses: the use of the extended CT-scale. Phys Med Biol 48:1591-1603

3 Oehler M, Buzug TM (2007) The λ-mlem algorithm: An iterative reconstruction technique for metal artifact reduction in CT imagesAdvances in Medical Engineering. Springer, pp 42-47

4 Hamill JJ, Brunken RC, Bybel B, DiFilippo FP, Faul DD (2006) A knowledge-based method for reducing attenuation artefacts caused by cardiac appliances in myocardial PET/CT. Phys Med Biol 51:2901-2918

5 Park HS, Hwang D, Seo JK (2016) Metal Artifact Reduction for Polychromatic X-ray CT Based on a Beam-Hardening Corrector. IEEE Trans Med Imaging 35:480-487

6 Kachelriess M, Watzke O, Kalender WA (2001) Generalized multi-dimensional adaptive filtering for conventional and spiral single-slice, multi-slice, and cone-beam CT. Med Phys 28:475-490

7 Abdoli M, Mehranian A, Ailianou A, Becker M, Zaidi H (2016) Assessment of metal artifact reduction methods in pelvic CT. Med Phys 43:1588-1597

8 Mehranian A, Ay MR, Rahmim A, Zaidi H (2013) X-ray CT metal artifact reduction using wavelet domain L0 sparse regularization. IEEE Trans Med Imaging 32:1707-1722

9 Mehranian A, Ay MR, Rahmim A, Zaidi H (2013) 3D Prior Image Constrained Projection Completion for X-ray CT Metal Artifact Reduction. IEEE Trans Nucl Sci 60:3318-3332

10 Weeks KJ, Montana GS (1997) Three-dimensional applicator system for carcinoma of the uterine cervix. Int J Radiat Oncol Biol Phys 37:455-463
